# Rationale and design of a longitudinal study of cerebral small vessel diseases, clinical and imaging outcomes in patients presenting with mild ischaemic stroke: Mild Stroke Study 3

**DOI:** 10.1177/2396987320929617

**Published:** 2020-06-05

**Authors:** Una Clancy, Daniela Jaime Garcia, Michael S Stringer, Michael J Thrippleton, Maria C Valdés-Hernández, Stewart Wiseman, Olivia KL Hamilton, Francesca M Chappell, Rosalind Brown, Gordon W Blair, Will Hewins, Emilie Sleight, Lucia Ballerini, Mark E Bastin, Susana Munoz Maniega, Tom MacGillivray, Kirstie Hetherington, Charlene Hamid, Carmen Arteaga, Alasdair G Morgan, Cameron Manning, Ellen Backhouse, Iona Hamilton, Dominic Job, Ian Marshall, Fergus N Doubal, Joanna M Wardlaw

**Affiliations:** Centre for Clinical Brain Sciences, University of Edinburgh, Edinburgh, UK

**Keywords:** Cerebral small vessel diseases, lacunar stroke, white matter hyperintensities, magnetic resonance imaging, longitudinal studies, dementia, cognitive dysfunction, symptom assessment, blood–brain barrier, cerebrovascular circulation

## Abstract

**Background:**

Cerebral small vessel disease is a major cause of dementia and stroke, visible on brain magnetic resonance imaging. Recent data suggest that small vessel disease lesions may be dynamic, damage extends into normal-appearing brain and microvascular dysfunctions include abnormal blood–brain barrier leakage, vasoreactivity and pulsatility, but much remains unknown regarding underlying pathophysiology, symptoms, clinical features and risk factors of small vessel disease.

**Patients and Methods:** The Mild Stroke Study 3 is a prospective observational cohort study to identify risk factors for and clinical implications of small vessel disease progression and regression among up to 300 adults with non-disabling stroke. We perform detailed serial clinical, cognitive, lifestyle, physiological, retinal and brain magnetic resonance imaging assessments over one year; we assess cerebrovascular reactivity, blood flow, pulsatility and blood–brain barrier leakage on magnetic resonance imaging at baseline; we follow up to four years by post and phone. The study is registered ISRCTN 12113543.

**Summary:**

Factors which influence direction and rate of change of small vessel disease lesions are poorly understood. We investigate the role of small vessel dysfunction using advanced serial neuroimaging in a deeply phenotyped cohort to increase understanding of the natural history of small vessel disease, identify those at highest risk of early disease progression or regression and uncover novel targets for small vessel disease prevention and therapy.

## Background

Cerebral small vessel disease (SVD) describes diffuse disease processes affecting the perforating cerebral arterioles, capillaries, venules and consequent damage to the white and deep grey matter.^1^ This damage is visible on brain MRI as white matter hyperintensities (WMH), recent small subcortical infarcts, perivascular spaces, brain atrophy and cerebral microbleeds.^2^

SVD causes 20% of ischaemic strokes and almost half of all dementias,^3,4^ contributing to both vascular and Alzheimer’s dementia subtypes,^5^ its presence more than doubling future risk of stroke, dementia and functional impairment.^6^

Recent advances in neuroimaging have uncovered candidate mechanisms for underlying pathophysiological processes. Furthermore, SVD appears to be more dynamic and global than previously thought, since recent studies show: (a) WMH can regress as well as progress;^7–10^ (b) SVD is associated with cerebrovascular dysfunction including diffuse blood–brain barrier (BBB) failure;^11^ (c) with some evidence for other vascular dysfunctions including reduced cerebrovascular reactivity (CVR) and increased intracranial pulsatility^12–14^ and (d) acute, apparently ‘silent’, lesions on diffusion weighted imaging (DWI) may be more frequent than previously thought.^15–17^

Most SVD lesions are thought to develop ‘silently’. However, some studies suggest that SVD lesions are associated with subjective cognitive complaints,^18^ gait disturbance,^19^ mood disorders and apathy.^20^ Moreover, subtle symptoms have been associated with acute DWI lesions in a few small cross-sectional studies in non-stroke populations (*n* = 6/649; *n* = 10/30),^21,22^ while apparently ‘silent’ acute DWI lesions have been noted in up to 25% following recent stroke, mostly in small studies (e.g. *n* < 105) that sought typical stroke symptoms.^16,23^ Thus, knowledge of the extent of clinical correlates of SVD lesions, in particular, any ‘red flag’ symptoms or signs that might highlight lesion worsening, remains limited and may be being clinically overlooked.

SVD is commonly attributed to traditional vascular risk factors, particularly hypertension, but also smoking and diabetes, yet these factors only account for 2% of WMH variance.^24^ Less is known about potential contributors such as diet, lifestyle and premorbid factors.^25^ Extending the search beyond an individual’s current clinical status to early and mid-life stages is an important target for SVD research.^26^ Understanding whether combined risk factors have a synergistic effect on an individual’s risk of developing SVD, as well as improved recognition of symptoms, would provide better recognition of persons at risk of SVD development or progression, providing insight on whether multimodal approaches to prevention and treatment should be taken.

Few studies have comprehensively assessed SVD lesion progression, symptomatology and wide-ranging risk factors. Hence we describe the protocol for a detailed study to assess the role of cerebrovascular dysfunctions in combination, symptoms, and risk factors including diet, sleep and early life factors, on longitudinal SVD lesion change in patients presenting with stroke-related SVD.

## Methods

### Study design

The Mild Stroke Study 3 (MSS-3: ISRCTN 12113543) is a detailed prospective observational cohort study with clinical and imaging follow-up which aims to recruit up to 300 participants. In addition to a scan at initial stroke diagnosis, the patients undergo a minimum of three study scans over a year; in addition, those with lacunar stroke or moderate-to-severe WMH are invited for a further one or two visits between baseline and six months. The baseline assessment occurs within a maximum of three months of index stroke (Table 1). All participants return 6 and 12 months, respectively, after the baseline assessment. Annually, thereafter up to four years, we will invite participants to continue annual postal or phone follow-up with another MRI at three years. The follow-up questionnaire includes recurrent vascular events, cognition and functional status. MSS-3 benefits from systems established during the MSS-1 and MSS-2 studies^27,28^ and commenced in August 2018.

**Table 1.** Study assessments.

Activity/assessment	Pre-visit	V1^a^: baseline	V2^b^: 2–3 months	V3^c^: 6 months	V4^d^: 12 months
Eligibility	X				
Diagnostic MRI/CT	X				
Consent		X			
Routine blood tests	X				
Electrocardiogram	X				
Carotid Doppler ultrasound	X				
Symptom assessment		X	X	X	X
Cognitive tests		X	X	X	X
MRI		X	X	X	X
Retinal imaging		X	X	X	X
Blood pressure		X	X	X	X
Recurrent vascular events		X	X	X	X
NIHSS		X	X	X	X
mRS		X		X	X
Medical history/vascular risk factors		X		X	X
Demographic/socioeconomic factors		X			
Diet questionnaire		X			
Sleep questionnaire		X			
Mood/fatigue questionnaires		X			
Blood/urine collection		X			
24-h blood pressure monitoring		X			
Premorbid IQ (education, National Adult Reading Test)		X			
Informant questionnaire: IQCODE, NPI-Q, AES-I		X			X
Pulse wave measures		X			
Nine-hole peg test		X			X
Timed up + go		X			X
Stroke Impact Scale				X	X

^a^Visit 1: baseline visit within three months of index stroke ±1 week.

^b^Visit 2: ±1 week.

^c^Visit 3: ±2 weeks.

^d^Visit 4: ±3 weeks.

MRI: magnetic resonance imaging; NIHSS: National Institute of Health Stroke Scale; IQCODE: Informant Questionnaire for Cognitive Decline in the Elderly; NPI-Q: Neuropsychiatric Inventory Questionnaire; AES-I: Apathy Evaluation Scale-Informant.

### Study population

Adults >18 years old with mild ischaemic stroke with a modified Rankin scale (mRS) ≤2 at recruitment presenting to Edinburgh/Lothian stroke services.

### Eligibility criteria

We define stroke as described previously:^27,28^ clinical lacunar stroke syndrome (50%) and control participants with non-lacunar ischaemic stroke syndromes (50%) i.e. partial anterior circulation syndrome or posterior circulation syndrome, with recent infarct visible on diagnostic MRI or CT scan compatible with the clinical syndrome, or if no visible infarct, no other lesion explaining the stroke symptoms. Participants with non-lacunar stroke form controls, since they have similar vascular risk factors and follow similar secondary prevention, accounting for medication effects on vessel function.

We exclude participants with MRI contraindications, major neurological conditions, severe cardiac and respiratory disease. All participants give written informed consent. The study was granted ethical approval by Southeast Scotland Regional Ethics Committee (reference 18/SS/0044).

### Diagnosis

An expert panel of stroke physicians and neuroradiologists reach final stroke diagnosis by consensus following review of presenting symptoms and signs including motor or sensory deficit, hemianopia, visuospatial disorder, ataxia, dysphasia, dysarthria, cerebellar or brainstem symptoms, supplemented by diagnostic brain MRI or CT and other relevant investigations, as previously described.^27,28^ An experienced neuroradiologist (JMW) assesses all scans for acute ischaemic lesions including recent small subcortical infarcts, prior infarcts or haemorrhages, WMH, lacunes, PVS, microbleeds, siderosis and atrophy, using standardised validated scales.^2,28^

### Vascular risk factors, past medical history, medications and incident vascular events

Each participant provides a medical history of diagnoses confirmed by a physician, supplemented by hospital medical records and general practitioner correspondence, following standard definitions, including diabetes mellitus, hypertension, hypercholesterolaemia, previous stroke or TIA, peripheral vascular disease, atrial fibrillation, ischaemic heart disease, valvular defects, heart failure and physician-diagnosed anxiety, depression or delirium. We record current medications, cross-checking with electronic medical records. At follow-up, we record recurrent stroke, TIA and cardiac events.

### Subjective symptoms

We use a structured questionnaire to ask open-ended questions about subjective symptoms experienced prior to, at the time of, and since index stroke diagnosis (see online Supplementary Appendix 1). Participants also answer questions based on previous clinico-radiological studies regarding symptoms within the past month including subjective memory concerns, confusional episodes, unsteadiness, falls, dizziness and headaches.^29^

Participants self-administer the Fatigue Severity Scale,^30^ Generalized Anxiety Disorder-7,^31^ the Center for Epidemiologic Studies-Depression Scale^32^ and an adapted Pittsburgh Sleep Quality Index.^33^

### Informant-reported symptoms

A nominated close friend or relative completes the following prior to the baseline visit: Neuropsychiatric Inventory Questionnaire,^34^ behavioural changes since stroke,^35^ Apathy Evaluation Scale, Informant version^36^ and the Informant Questionnaire for Cognitive Decline in the Elderly^37^ repeated at 12 months.

### Family, lifestyle, social and early life factors

We record stroke or dementia family history including age at diagnosis, alcohol consumption and smoking status including quantity and duration. Participants self-administer the EPIC-Norfolk Food Frequency Questionnaire, a comprehensive dietary overview including salt intake.^38,39^

To assess early life socioeconomic status, we record childhood postal address, number of individuals, rooms and toilets in the property and parental occupations. We note ethnicity, educational duration and attainment,^40^ occupation, current postcode and retirement age.

### Physical examination

We record presenting and current neurological deficits and stroke severity (NIHSS), blood pressure three times, gait (Timed Up and Go), manual dexterity (9-Hole Peg Test), height and weight.

### Cognitive assessment

Participants complete the comprehensive 30-min neuropsychological test protocol based on the National Institute of Neurological Disorders and Stroke–Canadian Stroke Network (NINDS-CSN) Vascular Cognitive Impairment Harmonization Standards. This battery spans multiple cognitive domains and includes the Montreal Cognitive Assessment (MoCA), Hopkins Verbal Learning Test-revised, Controlled Oral Word Association Test, Animal Naming, Letter Digit Coding and Trailmaking Tests A + B.

We estimate peak adult intelligence using the National Adult Reading Test.^40^

Participants repeat MoCA and Trailmaking Tests A + B at each visit. We use three different MoCA versions, randomly assigning a test sequence to each participant to minimise learning effects on serial test performance.

### Functional recovery

We administer the mRS^41^ at baseline, 6 and 12 months and the Stroke Impact Scale^42^ at 6 and 12 months.

### Magnetic resonance imaging

We scan all participants at diagnosis at 1.5 T (General Electric Signa HDxt) or 3 T (Siemens Prisma) MRI or CT with core structural brain MRI sequences at each visit: 3D T1w, T2w, fluid attenuated inversion recovery, susceptibility-weighted (SWI/SWAN/GRE) and single- or multi-shell diffusion imaging (dMRI). Subsequent full cerebrovascular assessment and all follow-up imaging are at 3 T.

At one to three months post-stroke, participants undergo 3 T MRI to measure BBB integrity, CVR, cerebral blood flow (CBF) and intracranial vascular and CSF pulsatility (protocol in online Supplementary Appendix 2). We assess BBB integrity using dynamic contrast-enhanced (DCE)-MRI and gadolinium-based contrast agent (gadobutrol) injection,^11,43^ unless eGFR <30 ml/min. We assess CVR using a blood oxygenation level dependent (BOLD) MRI sequence, during which participants inhale air with intermittent-added CO_2_ (12-min paradigm alternating 2 min air and 3 min 6% CO_2_) through a tight-fitting facemask, described previously.^13,44^ Arterial, venous and CSF pulsatility are measured using phase contrast MRI sequences.^14,44^ We measure CBF using major arterial phase contrast flow measures obtained during pulsatility measurements (and arterial spin labelling where feasible).

We process MRI computationally using well-validated methods to assess intracranial volume, CSF, normal-appearing white and grey matter, WMH volumes, index and prior stroke lesion volumes, lacunes, microbleeds and perivascular space metrics.^45,46^ We visually quantify index and prior stroke lesions (location, type), WMH (baseline, change), lacunes (number, location), perivascular spaces, microbleeds, siderosis, superficial and deep brain volume loss, according to STRIVE criteria using validated scales.^2,47–51^ See online Supplementary Appendix 2 for image processing methods description including advanced neuroimaging data.

### Retinal imaging

We assess vision (Logmar cabinet, Sussex Vision) and use Spectralis OCT2® with Optical Coherence Tomography Angiography (OCTA) (Heidelberg Engineering) at each visit, imaging retinal vessels, retinopathy, nerve fibre layer thickness, choroid OCTA, intra-retinal and sub-retinal fluid. We computationally process retinal and arteriolar widths, branching patterns, complexity,^52^ nerve fibre layer thickness and microvessels on OCTA using well-validated tools.^53^ See online Supplementary Appendix 2 for processing details.

### Systemic vascular measures

We record BP at three standard points during the baseline visit (Omron) and measure arterial velocities through pulse wave velocity and pulse wave analysis using a tonometric device (*SphygmoCor*®, AtCor Medical/Vicorder, Skidmore Medical) held over the carotid and radial pulses while supine. We provide a 24-h ambulatory BP monitoring device (SpaceLabs Medical) to most and encourage all to submit self-monitored blood pressure recordings. We repeat BP measurements at all study visits.

### Biochemical, haematological, cardiovascular and imaging investigations

We document routinely collected index stroke investigation results including serum haematology and biochemistry, electrocardiogram, echocardiography and carotid Doppler ultrasound. We collect 18 ml venous blood at baseline for inflammatory and endothelial function markers. We store 20 ml urine for inflammatory marker analysis and 5 ml for albumin-to-creatinine ratio.

## Endpoints

The primary endpoint is the proportion of SVD lesions that regress, progress or appear de novo in the year after stroke. The secondary endpoints are: (a) BBB integrity; (b) CVR; (c) intracranial vascular/CSF pulsatility; (d) WMH, PVS, lacunes and microbleeds; (e) white matter structural integrity measured with diffusion tensor and *T*_1_ parameters and (f) incidence of reported symptoms including neuropsychiatric and cognitive symptoms, recurrent stroke, transient ischaemic attacks and cardiac events.

### Statistical analysis

#### Sample size calculation

In a previous study with one year of longitudinal imaging follow-up at this centre, 10.6% of participants had a de novo lesion on MRI at follow-up.^28^ In the same study, 65% had a mean 5.5 ml WMH volume increase and 35% had a mean 6.6 ml decrease.^54^ A sample of 250 participants would be required to detect WMH change in the year after stroke, with significance 0.05 and power 0.90 in univariate analysis. We aim to recruit up to total 300 participants which allows for loss to follow-up.^54^

#### Proposed analyses

In our primary analyses, we will use linear mixed effects models adjusted for age, vascular risk factors and baseline SVD burden to estimate the effect of cerebrovascular structure/function on SVD lesion progression and regression.

Secondarily, we will use similar models and other approaches (e.g. stratifying by low vs. high SVD burden, stroke subtype) to quantify associations of the following factors with lesion change: cognitive test scores and incident cognitive impairment; symptom test scores and incident symptoms; functional status; life course factors; lifestyle factors; blood pressure; systemically measured vascular stiffness; retinal measures; and inflammatory and endothelial function markers.

## Discussion

The MSS-3 is a detailed prospective observational study which will advance our knowledge of how detailed measures of small vessel dysfunction and changing lesions relate to comprehensive symptom, cognitive, retinal, early life and lifestyle factors, whether some individuals are more vulnerable than others to the effects of small vessel dysfunction and whether a single candidate measure could best differentiate abnormal from normal-appearing brain tissue by stage and severity of SVD.

This study is novel in its concurrent use of advanced neuroimaging techniques to measure CVR, BBB leakage, CBF and vascular/CSF pulsatility: the first time these measures have been performed contemporaneously, alongside an unprecedented comprehensive assessment of symptoms and signs as they relate to these measures and lesion changes across multiple time-points. We build on previous studies,^8,55^ establishing a well-phenotyped profile of the dynamic natural history of SVD, capturing this rich dataset in the subacute post-stroke phase, monitoring the vulnerable brain at risk for early disease accumulation.^16,56^ Our systematic approach is a template for application to future research studies, designed to optimally assess rates of disease progression and regression, translatable to other SVD presentations including mild cognitive impairment.

This study will fill an existing gap of longitudinal imaging studies evaluating symptoms such as apathy, fatigue, anxiety, delirium, sleep disturbance and emotional liability, contributing to the detection of preclinical SVD states. We will identify ‘red flags’ to the presence and progression of SVD, so that we may intercept disease earlier, even before it develops, rather than in patients presenting with overt brain dysfunction, e.g. stroke, dementia. We will gain insight into novel preventative and therapeutic targets by uncovering the nature of and factors associated with lesion regression. This study will deepen our understanding of SVD, essential to future prevention and treatment of stroke and vascular dementia.

To date, we have recruited 116 participants. The baseline visit lasts 6.5 h, and most are willing to attend three further visits, each lasting two hours, with positive participant feedback, demonstrating the feasibility of applying this design at other centres.

## Trial registration

ISRCTN 12113543

## Supplemental Material

sj-pdf-1-eso-10.1177_2396987320929617 - Supplemental material for Rationale and design of a longitudinal study of cerebral small vessel diseases, clinical and imaging outcomes in patients presenting with mild ischaemic stroke: Mild Stroke Study 3 Click here for additional data file. Supplemental material, sj-pdf-1-eso-10.1177_2396987320929617 for Rationale and design of a longitudinal study of cerebral small vessel diseases, clinical and imaging outcomes in patients presenting with mild ischaemic stroke: Mild Stroke Study 3 by Una Clancy, Daniela Jaime Garcia, Michael S Stringer, Michael J Thrippleton, Maria C Valdés-Hernández, Stewart Wiseman, Olivia KL Hamilton, Francesca M Chappell, Rosalind Brown, Gordon W Blair, Will Hewins, Emilie Sleight, Lucia Ballerini, Mark E Bastin, Susana Munoz Maniega, Tom MacGillivray, Kirstie Hetherington, Charlene Hamid, Carmen Arteaga, Alasdair G Morgan, Cameron Manning, Ellen Backhouse, Iona Hamilton, Dominic Job, Ian Marshall, Fergus N Doubal and Joanna M Wardlaw in European Stroke Journal

sj-pdf-2-eso-10.1177_2396987320929617 - Supplemental material for Rationale and design of a longitudinal study of cerebral small vessel diseases, clinical and imaging outcomes in patients presenting with mild ischaemic stroke: Mild Stroke Study 3 Click here for additional data file. Supplemental material, sj-pdf-2-eso-10.1177_2396987320929617 for Rationale and design of a longitudinal study of cerebral small vessel diseases, clinical and imaging outcomes in patients presenting with mild ischaemic stroke: Mild Stroke Study 3 by Una Clancy, Daniela Jaime Garcia, Michael S Stringer, Michael J Thrippleton, Maria C Valdés-Hernández, Stewart Wiseman, Olivia KL Hamilton, Francesca M Chappell, Rosalind Brown, Gordon W Blair, Will Hewins, Emilie Sleight, Lucia Ballerini, Mark E Bastin, Susana Munoz Maniega, Tom MacGillivray, Kirstie Hetherington, Charlene Hamid, Carmen Arteaga, Alasdair G Morgan, Cameron Manning, Ellen Backhouse, Iona Hamilton, Dominic Job, Ian Marshall, Fergus N Doubal and Joanna M Wardlaw in European Stroke Journal
