# Supplementary material for: Rationale and design of a longitudinal study of cerebral small vessel diseases, clinical and imaging outcomes in patients presenting with mild ischaemic stroke: Mild Stroke Study 3
Source: Eur Stroke J. 2020 Jun 5;6(1):81–8. doi: 10.1177/2396987320929617 (PMC7995323; doi:10.1177/2396987320929617)
Supplement: sj-pdf-1-eso-10.1177_2396987320929617 - Supplemental material for Rationale and design of a longitudinal study of cerebral small vessel diseases, clinical and imaging outcomes in patients presenting with mild ischaemic stroke: Mild Stroke Study 3 [file sj-pdf-1-eso-10.1177_2396987320929617.pdf]

Title: Studies of small vessel diseases: the Mild Stroke Study 3 (MSS-3). The longitudinal study of cerebral small vessel diseases following mild ischaemic stroke: rationale and design.

Supplement 2:

(a) MRI protocol at baseline assessment

(b) Summary of image analysis methods

(a) MRI protocol at baseline assessment

	MRA	Flow			quantitative T_1			DCE-MRI
**Sequence**	**TOF**	**2D PC (carotids)**	**2D PC (SACSF)**	**2D PC (sinus)**	**3D IR-sGRE (TI = 600 ms)**	**3D IR-sGRE (TI = 1500 ms)**	**3D sGRE (FA = 2°,5°, 12°)**	***T*1w 3D sGRE**
**Voxel size (mm)**	0.5x0.7x1.6	1.0x1.0x5.0	0.8x0.8x5.0	0.7x0.7x5.0	1.2x1.2x1.2	1.2x1.2x1.2	1.2x1.2x1.2	2x2x2
**TR (ms)**	20.0	19.60	25.18	21.70	1040	1940	5.4	3.44
**TE (ms)**	3.51	5.82	8.45	6.59	1.82	1.82	1.82	1.68
**TI**	-	-	-	-	600	1500	-	-
**Flip Angle (°)**	20	12	12	12	5	5	2, 5, 12	15
**Acquisition Time (mm:ss)**	2:45	1:39 approx.	1:55 approx.	2:11 approx.	1:55	3:35	1:36 x 3	21:08
**Other**		*R*=2<br>venc = 70 cm s^{-1}<br>NA = 2	*R*=2<br>venc = 6 cm s^{-1}	*R*=2<br>venc = 50 cm s^{-1}	*R*=2	*R*=2	*R*=2	32 volumes

	CVR	T1w	FLAIR	PD	T2w	SWI	dMRI	ASL
**Sequence**	**2D GE-EPI**	**MPRAGE (3D IR-sGRE)**	**SPACE (3D RARE)**	**3D sGRE**	**SPACE (3D RARE)**	**3D sGRE**	**2D GE-EPI**	**3D pcASL**
**Voxel size**	2.5x2.5x2.5	1.0x1.0x1.0	1.0x1.0x1.0	1.0x1.0x1.0	0.9x0.9x0.9	0.6x0.6x3.0	2.0x2.0x2.0	3.4x3.5x3.5
**TR**	1550	2500	5000	6.04	3200	28	4300	4350
**TE**	30.0	4.37	388	2.44	408	20	74.0	20.98
**TI**	-	-	1100	1800	-	-	-	-
**Flip Angle**	67	7	-	2.0	-	9	-	-
**Acquisition time**	12:30	3:45	5:57	1:57	3:42	4.02	11:16	3:45
**Other**	*R*=2, *MB*=2	*R*=3	*R=3*	*R*=3	*R*=2x2	*R*=2	*R*=2, *MB*=2<br>15 × b = 0 s/mm2, 3 × b = 200 s/mm2, 6 × b =	*R*=2<br>*TI* = 500-

			TI=1800 ms				500 s/mm2, 64 × b = 1000 s/mm2, 64 × b = 2000 s/mm2<br>(3 x b0 acquired with reversed phase ecoding)	3030 (x12)

CVR =Cerebrovascular reactivity; MPRAGE =Magnetization-prepared rapid acquisition with gradient echo; FLAIR =Fluid-attenuated inversion recovery; PD VIBE =Proton density; SPACE =Sampling perfection with application-optimized contrast using different flip-angle evolution; SWI =Susceptibility weighted imaging; dMRI =Diffusion imaging; pcASL =Pseudo-continuous arterial spin labelling; TOF =Time-of-flight; PC =Phase-contrast; SACSF =Subarachnoid cerebrospinal fluid; IR =Inversion recovery; sGRE =Spoiled gradient recalled echo; TI =Inversion time; FA =Flip angle; DCE =Dynamic contrast-enhanced; TR =Repetition time; TE =Echo time; *R*=parallel imaging acceleration factor; *MB*=multiband acceleration factor; NA = number of averages.

(b) Summary of image analysis methods

Structural and Diffusion Imaging

The index, old and recurrent infarcts and SVD imaging markers (i.e. white matter hyperintensities (WMH), lacunes, perivascular spaces (PVS) and microbleeds are assessed by an expert neuroradiologist using validated visual scores (1-4), and recorded in standard assessment templates (5, 6) as described previously.(7)

All images are converted from DICOM to NIFTI-1 format using dcm2niix (https://github.com/rordenlab/dcm2niix). For each patient, structural tissue/lesion segmentation is performed in the native space of the T2-weighted image acquired at visit 1. Therefore, we linearly align all structural sequences from all visits to this image space using FSL-FLIRT (8). The structural processing pipeline is fully automatic and combines the output from state-of-the-art neuroimaging processing tools: FSL-FAST (9), freesurfer

(https://surfer.nmr.mgh.harvard.edu/), LOTS-IM (10) and multispectral Gaussian clustering optimised using an Expectation-Maximisation algorithm (11) to output the volumes, probabilistic and binary masks of: 1) venous sinuses, meninges and main venous pathways, 2) cerebrospinal fluid, 3) intense and less-intense WMH, 4) normal-appearing white matter 5) deep grey matter structures, 6) cortical grey matter, 7) stroke lesions and 8) lacunes, total and per cerebral and cerebellar hemisphere. PVS are segmented in the native T2W space in the basal ganglia and centrum semiovale regions for each visit as described previously (2, 12), both segmented fully automatically using the output from the main structural pipeline. Venous pathways and mineral deposition are segmented in the native SWI space using the minimum intensity projection, phase and magnitude images combined with the T1w sequence.(13, 14)

Diffusion data are processed using TractoR version 3.3.(15) DICOM data are converted to NIfTI-1 format using 'divest' (16), corrected for susceptibility and eddy current induced distortions using topup and eddy from FSL version 6.0.1 (17-19), and the brain is masked using FSL's brain extraction tool. The water self-diffusion tensor is calculated for each brain voxel, and parametric maps of fractional anisotropy (FA) and mean diffusivity (MD) are derived from its eigenvalues with TractoR's 'tensorfit' using an iterative weighted least-squares approach.(20) NODDI parameters (intracellular volume fraction (ICVF), isotropic volume fraction (ISOVF) and orientation dispersion index (ODI)) will be determined from the registered multi-shell diffusion MRI data using the NODDI Matlab toolbox (http://mig.cs.ucl.ac.uk/).

Advanced imaging measures of vessel function

Blood brain barrier permeability

Full details of the DCE-MRI acquisition protocol are available to download at the Harmonizing Brain Imaging Method for Vascular Contributions to Neurodegeneration (HARNESS) website (21): https://harness-neuroimaging.org

In summary, we derive values for the blood plasma volume fraction (v_P) and the capillary permeability-surface area product *PS* for each voxel and region as described in (22).

Cerebrovascular flow, perfusion, and reactivity

The multi-inversion time pseudo-continuous arterial spin labelling data is processed through FSL's BASIL using a 1-compartment model and partial volume correction to obtain cerebral blood flow and arterial transit time (12 equally spaced TIs=500-3030ms, TR/TE=4350/20.98ms with 4 background suppression pulses, bolus duration=1800ms).(23) Further analysis is performed using white and subcortical grey matter regions of interest.

We acquire four phase-contrast scans following manual placement of a 2D slice perpendicular to the following vessels before manual segmentation: internal carotid and vertebral arteries, internal jugular veins, venous sinuses (superior sagittal, straight, and transverse sinuses), subarachnoid CSF at the level of C2-C3 and aqueduct. We manually segment vessel regions of interest (ROIs) using FSLeyes before processing phase-contrast MRI data, using in-house MATLAB code to obtain flow measurements.

We extract velocity on a pixel-by-pixel basis and calculate the blood/CSF flow for each vessel/space, performing aliasing and background corrections where required. We calculate flow across the cardiac cycle for each vessel and estimate the pulsatility index (PI = (max

flow - min flow) / mean flow). We also extract the pulse waveform delay between the carotids and other intracranial vessels.(24)

During the 12 minute cerebrovascular reactivity (CVR) paradigm using a Blood Oxygenation Level Dependent (BOLD) MRI, we record end-tidal CO2 (ETCO2) while participants alternately inhale medical air (2 minutes) and air containing 6% carbon dioxide (3 minutes). Linear regression with a variable CVR delay is used to extract measurements of cerebrovascular reactivity (% change in BOLD signal per mmHg change in ETCO2) and the delay value in white and grey matter.(25)

Retinal imaging

All retinal images are acquired with a SPECTRALIS imaging platform (Heidelberg Engineering, Heidelberg, Germany) that combines fundus imaging with a scanning laser ophthalmoscope and simultaneous optical coherence tomography (OCT) imaging. The camera employs spectral domain OCT which achieves micrometre resolution with very fast scanning times. The beam of a super luminescence diode scans across the retina to produce cross-sectional images, with an infrared wavelength of 870nm.

The retinal imaging protocol builds on systems successfully established during the Mild Stroke Study 1 at our centre.(26, 27) Both eyes are imaged during a 25minute protocol that includes: horizontal and vertical single line scans through the macula, enhanced depth imaging (EDI) to permit enhanced visualisation and subsequent measurement of the sub-foveal choroidal thickness, posterior pole multi-line imaging that captures 61 individual slices inferiorly to superiorly across the retina, circular optic nerve head scan for vascular

assessment of main vessels and peripapillary retinal nerve fibre layer (RNFL) thickness measurements, and multicolour imaging via three colour wavelengths for an assessment of maculopathy and retinopathy. Additionally, we conduct optical coherence tomography angiography (OCTA) to assess the microvasculature, including vessel area density of the superficial vascular complex which supplies the RNFL and the ganglion cell layer.

The retinal images are used as input to two themes of analysis: vascular and neuroretinal. Fundus images are processed with the Vascular Assessment and Measurement Platform for Images of the REtina (VAMPIRE; Web version, Universities of Edinburgh and Dundee: vampire.computing.dundee.ac.uk), a validated software application for semi-automatic quantification of retinal vessel properties. RNFL segmentation is undertaken using the manufacturer's software. Vessel density of the small vessels discerned by OCTA is undertaken with bespoke image analysis software. We also administer a short ocular health questionnaire, assess visual acuity and measure eye axial length.

References

1. Fazekas F, Niederkorn K, Schmidt R, Offenbacher H, Horner S, Bertha G, et al. White matter signal abnormalities in normal individuals: correlation with carotid ultrasonography, cerebral blood flow measurements, and cerebrovascular risk factors. Stroke. 1988;19(10):1285-8.
2. Potter GM, Chappell FM, Morris Z, Wardlaw JM. Cerebral perivascular spaces visible on magnetic resonance imaging: development of a qualitative rating scale and its observer reliability. Cerebrovascular diseases (Basel, Switzerland). 2015;39(3-4):224-31.
3. Cordonnier C, Potter GM, Jackson CA, Doubal F, Keir S, Sudlow CL, et al. improving interrater agreement about brain microbleeds: development of the Brain Observer MicroBleed Scale (BOMBS). Stroke. 2009;40(1):94-9.
4. Penke L, Valdes Hernandez MC, Maniega SM, Gow AJ, Murray C, Starr JM, et al. Brain iron deposits are associated with general cognitive ability and cognitive aging. Neurobiology of aging. 2012;33(3):510-7.e2.
5. Association between brain imaging signs, early and late outcomes, and response to intravenous alteplase after acute ischaemic stroke in the third International Stroke Trial (IST-3): secondary analysis of a randomised controlled trial. The Lancet Neurology. 2015;14(5):485-96.
6. Wardlaw JM, Sellar R. A simple practical classification of cerebral infarcts on CT and its interobserver reliability. AJNR American journal of neuroradiology. 1994;15(10):1933-9.
7. Valdes Hernandez Mdel C, Armitage PA, Thrippleton MJ, Chappell F, Sandeman E, Munoz Maniega S, et al. Rationale, design and methodology of the image analysis protocol for studies of patients with cerebral small vessel disease and mild stroke. Brain and behavior. 2015;5(12):e00415.

8. Jenkinson M, Bannister P, Brady M, Smith S. Improved optimization for the robust and accurate linear registration and motion correction of brain images. NeuroImage. 2002;17(2):825-41.
9. Zhang Y, Brady M, Smith S. Segmentation of brain MR images through a hidden Markov random field model and the expectation-maximization algorithm. IEEE transactions on medical imaging. 2001;20(1):45-57.
10. Rachmadi MF, Valdes-Hernandez MDC, Li H, Guerrero R, Meijboom R, Wiseman S, et al. Limited One-time Sampling Irregularity Map (LOTS-IM) for Automatic Unsupervised Assessment of White Matter Hyperintensities and Multiple Sclerosis Lesions in Structural Brain Magnetic Resonance Images. Computerized medical imaging and graphics : the official journal of the Computerized Medical Imaging Society. 2019;79:101685.
11. McLachlan GJP, David. . Finite mixture models. New York ; Chichester : Wiley. 2000.
12. Ballerini L, Lovreglio R, Valdes Hernandez MDC, Ramirez J, MacIntosh BJ, Black SE, et al. Perivascular Spaces Segmentation in Brain MRI Using Optimal 3D Filtering. Scientific reports. 2018;8(1):2132.
13. A G. Characterisation and segmentation of basal ganglia mineralization in normal ageing with multimodal structural MRI. PhD Thesis. University of Edinburgh. . 2016.
14. Glatz A, Bastin ME, Kiker AJ, Deary IJ, Wardlaw JM, Valdes Hernandez MC. Automated segmentation of multifocal basal ganglia T2*-weighted MRI hypointensities. NeuroImage. 2015;105:332-46.
15. Clayden JD MMS, Storkey AJ, King MD, Bastin ME, Clark CA. TractoR: Magnetic Resonance Imaging and Tractography with R. J Stat Softw 2011:44:1–18. Available at: http://www.tractor-mri.org.uk/references.
16. Clayden JD RC. divest: Get images out of DICOM format quickly. 2017;Available at: https://cran.r-project.org/package=divest.
17. Andersson JL, Skare S, Ashburner J. How to correct susceptibility distortions in spin-echo echo-planar images: application to diffusion tensor imaging. NeuroImage. 2003;20(2):870-88.
18. Andersson JLR, Sotiropoulos SN. An integrated approach to correction for off-resonance effects and subject movement in diffusion MR imaging. NeuroImage. 2016;125:1063-78.
19. Smith SM, Jenkinson M, Woolrich MW, Beckmann CF, Behrens TE, Johansen-Berg H, et al. Advances in functional and structural MR image analysis and implementation as FSL. NeuroImage. 2004;23 Suppl 1:S208-19.
20. Salvador R, Pena A, Menon DK, Carpenter TA, Pickard JD, Bullmore ET. Formal characterization and extension of the linearized diffusion tensor model. Hum Brain Mapp. 2005;24(2):144-55.
21. Smith EE, Biessels GJ, De Guio F, de Leeuw FE, Duchesne S, During M, et al. Harmonizing brain magnetic resonance imaging methods for vascular contributions to neurodegeneration. Alzheimer's & dementia (Amsterdam, Netherlands). 2019;11:191-204.
22. Heye AK, Thrippleton MJ, Armitage PA, Valdes Hernandez MDC, Makin SD, Glatz A, et al. Tracer kinetic modelling for DCE-MRI quantification of subtle blood-brain barrier permeability. NeuroImage. 2016;125:446-55.
23. Chappell MA GA, Whitcher B, Woolrich MW. Variational Bayesian inference for a nonlinear forward model. IEEE Trans Signal Process. IEEE Trans Signal Process 2009;;57(1):223-236.
24. Shi Y, Thrippleton MJ, Blair GW, Dickie DA, Marshall I, Hamilton I, et al. Small vessel disease is associated with altered cerebrovascular pulsatility but not resting cerebral blood flow. Journal of cerebral blood flow and metabolism : official journal of the International Society of Cerebral Blood Flow and Metabolism. 2020;40(1):85-99.
25. Thrippleton MJ, Shi Y, Blair G, Hamilton I, Waiter G, Schwarzbauer C, et al. Cerebrovascular reactivity measurement in cerebral small vessel disease: Rationale and reproducibility of a protocol for MRI acquisition and image processing. International journal of stroke : official journal of the International Stroke Society. 2018;13(2):195-206.

26. Doubal FN, MacGillivray TJ, Hokke PE, Dhillon B, Dennis MS, Wardlaw JM. Differences in retinal vessels support a distinct vasculopathy causing lacunar stroke. Neurology. 2009;72(20):1773-8.
27. Doubal FN, MacGillivray TJ, Patton N, Dhillon B, Dennis MS, Wardlaw JM. Fractal analysis of retinal vessels suggests that a distinct vasculopathy causes lacunar stroke. Neurology. 2010;74(14):1102-7.
