# Supplementary material for: Rationale and design of a longitudinal study of cerebral small vessel diseases, clinical and imaging outcomes in patients presenting with mild ischaemic stroke: Mild Stroke Study 3
Source: Eur Stroke J. 2020 Jun 5;6(1):81–8. doi: 10.1177/2396987320929617 (PMC7995323; doi:10.1177/2396987320929617)
Supplement: sj-pdf-2-eso-10.1177_2396987320929617 - Supplemental material for Rationale and design of a longitudinal study of cerebral small vessel diseases, clinical and imaging outcomes in patients presenting with mild ischaemic stroke: Mild Stroke Study 3 [file sj-pdf-2-eso-10.1177_2396987320929617.pdf]

Title: Studies of small vessel diseases: the Mild Stroke Study 3 (MSS-3). The longitudinal study of cerebral small vessel diseases following mild ischaemic stroke: rationale and design.

Supplement 1: Symptom questionnaire

OTHER SYMPTOMS

Q45 (a) Other than the symptoms that first brought you to medical attention with your stroke, did you have any *other* symptoms *in the month prior to* the stroke?

Yes / No / Unknown

If "no", go to Q46

(b) If yes, please describe:

Symptom 1____

Symptom 2(optional)
____

Symptom 3(optional)
____

(c) Categorise domain of symptoms under the following:
Symptom 1 Gait / Low mood / Anxiety / Cognition / Apathy / Sleep and fatigue / Urinary / Sensory / Motor / Visual / Speech / Other

Symptom 2 (optional): Gait / Low mood / Anxiety / Cognition / Apathy / Sleep and fatigue / Urinary / Sensory / Motor / Visual / Speech / Other

Symptom 3 (optional): Gait / Low mood / Anxiety / Cognition / Apathy / Sleep and fatigue / Urinary / Sensory / Motor / Visual / Speech / Other

(d) Categorise characteristics of symptoms under the following:
Symptom 1:
Sudden onset / gradual onset /unknown
Duration <24hours / duration >24hours /unknown
Focal / non-focal /unknown
Resolved / ongoing /unknown

Symptom 2 (optional):
Sudden onset / gradual onset /unknown
Duration <24hours / duration >24hours /unknown

Focal / non-focal /unknown
Resolved / Ongoing /unknown

Symptom 3:
Sudden onset / gradual onset /unknown
Duration <24hours / duration >24hours /unknown
Focal / non-focal /unknown
Resolved / Ongoing /unknown

Q 46 (a) Other than the symptoms that first brought you to medical attention, have you had any *other* symptoms *since* the stroke?

Yes / No / Unknown

If "no", go to Q47

(b) If yes, please describe:

Symptom 1____

Symptom 2(optional) ____

Symptom 3(optional) ____

(c) Categorise domain of symptoms under the following:

Symptom 1 Gait / Low mood / Anxiety / Cognition / Apathy / Sleep and fatigue / Urinary / Sensory / Motor / Visual / Speech / Other

Symptom 2 (optional): Gait / Low mood / Anxiety / Cognition / Apathy / Sleep and fatigue / Urinary / Sensory / Motor / Visual / Speech / Other

Symptom 3 (optional): Gait / Low mood / Anxiety / Cognition / Apathy / Sleep and fatigue / Urinary / Sensory / Motor / Visual / Speech / Other

(d) Categorise characteristics of symptoms under the following:
Symptom 1:
Sudden onset / gradual onset /unknown
Duration <24hours / duration >24hours /unknown
Focal / non-focal /unknown
Resolved / ongoing /unknown

Symptom 2 (optional):
Sudden onset / gradual onset /unknown
Duration <24hours / duration >24hours /unknown

Focal / non-focal /unknown
Resolved / Ongoing /unknown

Symptom 3:
Sudden onset / gradual onset /unknown
Duration <24hours / duration >24hours /unknown
Focal / non-focal /unknown
Resolved / Ongoing /unknown

Q 47 Have you had any previous episodes of delirium?
(Select "No" if the following criteria are met: diagnosis is not recorded anywhere on TRAK correspondence *or* ECS *and* the patient has never been informed by a healthcare professional that they have got the diagnosis that is listed)

Yes /
No /
Unknown

Q 48 Do you have any concerns about your memory?

Yes /
No /
Unknown

Q49 Have you experienced a feeling of "brain fog" or lack of clarity in thinking during the past month?

Yes /
No /
Unknown

Q50 Have you experienced any episodes of confusion or felt confused during the past month?
Yes /
No /
Unknown

Q51 Have you felt unsteady on your feet during the past month?

Yes /
No /
Unknown

Q 52 Have you experienced any light-headedness, dizziness, vertigo, or any combination of the above during the past month

(a)
Yes /

No /
Unknown

(b) If yes, did you experience:
1- light-headedness
2- dizziness
3- vertigo
4- a combination of any of the above

Q53 Have you had any falls in the past month? (Note to interviewer: a fall is defined as an event which results in a person coming to rest inadvertently on the ground or floor or other lower level)

Yes /
No /
Unknown

Q54 During the last 30 days, on how many of these days did you have a headache? (answer 0 if none)

_________/unknown
